# Coupled changes in soil organic carbon fractions and microbial community composition in urban and suburban forests

**DOI:** 10.1038/s41598-020-73119-8

**Published:** 2020-09-28

**Authors:** Xueying Zhang, Xiaomei Chen, Muying Liu, Zhanying Xu, Hui Wei

**Affiliations:** 1 School of Geographical Sciences, Guangzhou University, 230 Wai Huan Xi Road, Guangzhou Higher Education Mega Center, Guangzhou, 510006 China; 2 College of Natural Resources and Environment, South China Agricultural University, Guangzhou, 510642 China

**Keywords:** Carbon cycle, Ecology, Climate change

## Abstract

Climate change and rapid urbanization have greatly impacted urban forest ecosystems and the carbon (C) cycle. To assess the effects of urbanization on forest soil C and soil microorganisms, six natural forests in a highly-urbanized region were selected as the research objects. Soil samples were collected to investigate the content and fractions of the soil organic carbon (SOC), as well as the soil microbial community composition. The results showed that the SOC content and fractions were substantially lower in the urban forests than in the suburban forests. Meanwhile, the total amount of phospholipid fatty acids (PLFAs) at suburban sites was twice more than that at urban sites, with shifts in microbial community structure. The potential differences in C inputs and nutrient limitation in urban forests may aggravate the low quantity and quality of SOC and consequently impact microbial community abundance and structure. Variation in microbial community structure was found to explain the loss of soil C pools by affecting the C inputs and promoting the decomposition of SOC. Therefore, the coupled changes in SOC and soil microorganisms induced by urbanization may adversely affect soil C sequestration in subtropical forests.

## Introduction

The forest SOC pool is regarded as one of the most important parts of the global C cycle; moreover, the urban forest ecosystem plays an important role in the regional C sink^1,2^. Studies show that urban forests are facing serious environmental problems, such as habitat destruction and fragmentation^3^, which is why we focus on remnant forests. Compared with those in suburbs, urban remnant forests and the soil in these forests are subject to greater impacts of climate change, such as global warming, elevated CO_2_, high atmospheric N deposition, and air pollution^4–6^. The formation of the urban heat island phenomenon is one of the most common impacts of urban expansion along with land cover change and forest degradation, which increase the temperature in urban areas relative to surrounding areas, namely, suburbs and rural areas^7^. In addition, vehicle exhaust and building energy consumption in central urban areas are the largest sources of greenhouse gases^8^. A study indicated that climate change will shift the forest ecosystems C cycle by changing the aforementioned environmental factors^2^. Paying close attention to remnant forest ecosystem with different degrees of urbanization and observing their responses to climate change can help predict the possible future impacts of persistent climate change on forest ecosystems^9^.

Environmental change can significantly influence forest soil C storage and its stability, especially in highly-urbanized regions. For example, the warming-induced stimulation of plant-derived C influx may offset the increased efflux in soil respiration or dissolved organic C leaching^10^. Many experimental studies illustrated that elevated CO_2_ levels would potentially enhance C sequestration in forest soils^1,11^; however, C sequestration would probably only increase very slightly but not in the long run depending on N availability^12^. It is noteworthy that the multiple factors of global climate change cannot be explained clearly using single-factor experiments. Although some studies have focused on interactive influences^13,14^, little research has revealed the long-term effects. Accordingly, studies using the urban–rural gradient approach would more closely reflect the actual change in C sequestration driven by climate change in the highly-urbanized region for a longer time scale.

SOC is described as a C continuum in varying states of decomposition and is affected by site conditions and biological limitations^15,16^. As reported in previous studies, SOC has been subdivided into active fractions and inert fractions according to their different turnover times and different functional C pools^17^. Labile SOC fractions, such as light fraction organic C, readily oxidizable organic C (ROC), water-soluble organic C, and microbial biomass C, are more sensitive than total organic C in response to environmental changes^18,19^; conversely, nonlabile SOC fractions (represented by non-readily oxidizable organic C (NROC) content in this study) are more closely related to SOC stability, especially in relatively active surface soil, in the early decomposition process of soil C^20^. Therefore, we calculated the changing proportions of SOC fractions to clarify soil C dynamics.

Additionally, Schmidt et al.^21^ proposed that molecular structure alone does not control soil organic matter (SOM) stability, and environmental conditions and soil microbial mechanisms are the most important factors^22^. The vegetation community, soil microenvironment, soil characteristics, etc. affect the decomposition of SOC via soil microbial mechanisms^23,24^. It has also been recognized that microbially derived compounds are the largest contributor to stable SOM^20,25^. Therefore, it is necessary to highlight the importance of microorganisms in studying SOC accumulation and stability. Urbanization-induced soil micro-ecological environmental changes have significant effects on soil C sequestration and soil microbial community structure^26,27^. However, the correlation between SOC and microbial community composition has rarely been analyzed in this respect, especially in subtropical forest ecosystems.

Subtropical forests are considered to be potentially large C sinks^28,29^. The characteristics of the vegetation community, nitrogen cycle, soil C pool, soil uptake potential for greenhouse gases, soil heavy metal pollution, soil microbes, etc. in urban remnant forests of the southern subtropical monsoon climate zone have changed in the highly-urbanized Pearl River Delta (PRD) region^6,30–35^. When studying *Pinus massoniana* forests in South China, Chen et al.^30^ indicated that the soil C pools were lower in urban sites because of the significant decrease in fine root biomass and a potential increase in SOC decomposition. In contrast, Groffman et al.^36^ studied the soil C pool along the urban–rural gradient forests in New York and found that the total C pool in the urban areas was higher; the labile SOC fractions decreased, but the nonlabile SOC fractions increased, resulting in a decrease in soil microbial biomass. Similarly, the research of Koerner and Klopatek^37^ undertaken in this region verified that urban forest soils have higher SOC, total nitrogen (TN), and nutrient contents. It can be inferred that the changes in the above variances were due to the differences in the input or output environments of soil C among different forest ecosystems. Thus, more experimental evidence should be provided to research how urbanization-induced environmental changes affect C sequestration in subtropical forests.

In this study, a contrast experiment was performed in the southern subtropical evergreen broadleaved forest area to assess the potential effects of urbanization on forest soil C and soil microorganisms. The remnant forests are divided into urban forests and suburban forests according to their distances from the urban center; meanwhile, land use and characteristics of the ecological environments in remnant forests were also taken into consideration. First, we hypothesized that the soil C content would decrease under the influence of urbanization even without direct disturbance and consequently change the soil microbial attributes^5,27^. Furthermore, we attempted to confirm whether the altered relative proportions of SOC fractions could lead to a reduction in SOC content^36,38,39^. It was also expected that the soil microbial community structure would change due to the correlations between SOC and soil microbes^23,24^. Moreover, the surface and subsurface soil layers would respond differently to environmental changes owing to their different physicochemical properties.

## Results

### Changes in SOC fractions and lability

The two-way ANOVA results showed that sampling site and soil depth significantly affected the SOC content and SOC fractions, including ROC and NROC (*p* < 0.05, Table 1), whereas the soil C lability was not significantly different among treatments (*p* > 0.05). The interactive effect of site and soil layer was not significant in the investigated SOC fractions and lability (*p* > 0.05 for all, Table 1), suggesting that between-site SOC changes in the urban versus suburban sites were consistent between the two investigated soil layers.

**Table 1:** Summary of the two-way ANOVA on soil organic carbon (SOC) fractions and soil microbial PLFA abundances.

	Site		Soil layer		Interaction	
	*F*	*p*	*F*	*p*	*F*	*p*
SOC	55.200	< 0.001	94.358	< 0.001	1.637	0.173
C lability	2.343	0.059	0.590	0.447	0.170	0.972
ROC	25.530	< 0.001	51.003	< 0.001	0.749	0.592
NROC	52.817	< 0.001	85.263	< 0.001	1.649	0.169
PLFA	31.065	< 0.001	46.848	< 0.001	3.267	0.022

C lability represents the ratio of ROC to NROC. ROC represents readily oxidizable organic C, and NROC represents non-readily oxidizable organic C. Distance from the urban center and soil layer were the two fixed factors. The *F*-statistics (*F*) and significance levels (*p*) are presented in the table.

**Figure 1:**
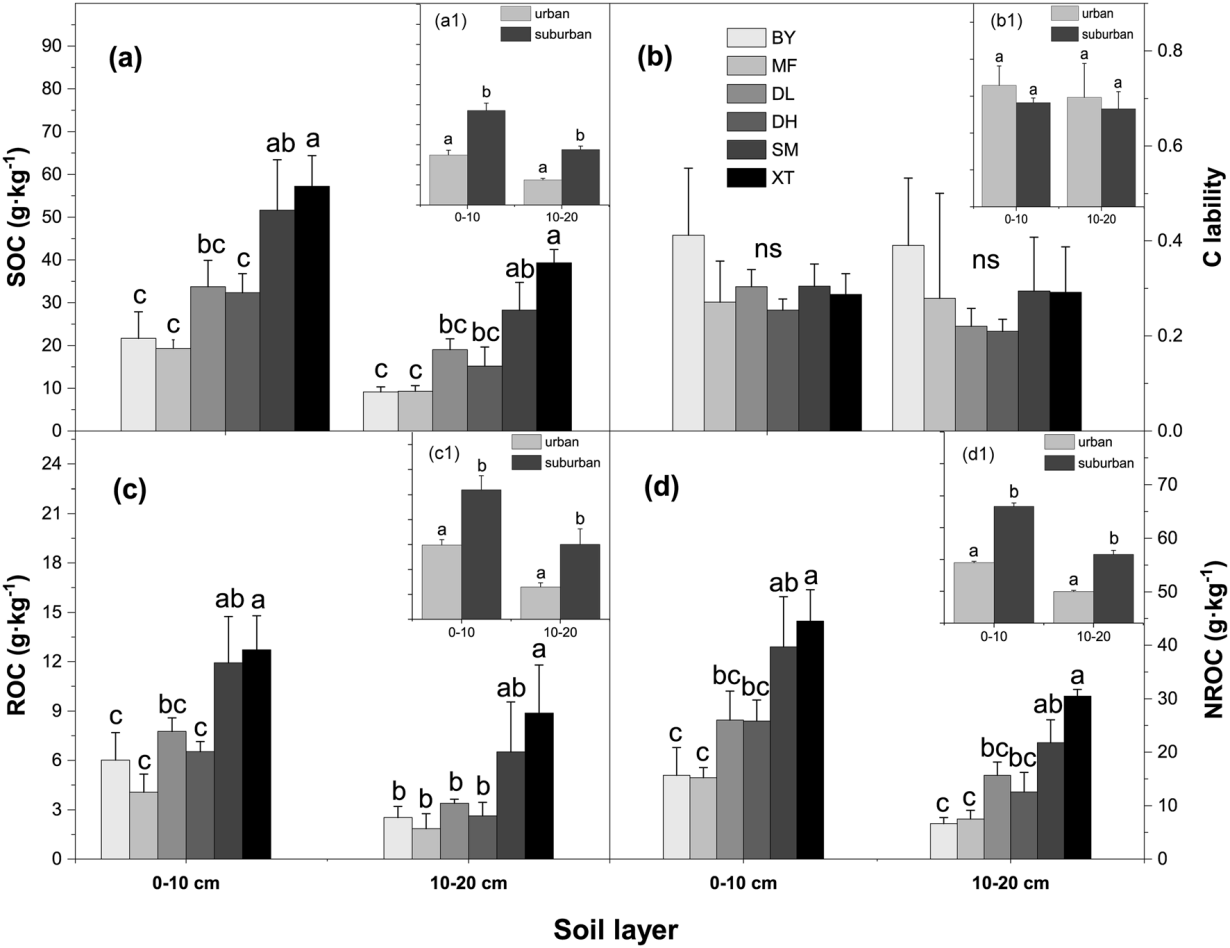
The contents of SOC and its fractions in the two soil layers. (**a**) Total soil organic C (SOC), (**c**) readily oxidizable organic C (ROC), (**d**) non-readily oxidizable organic C (NROC) concentrations, and (**b**) C lability (representing ROC/NROC) in every forest site in the two soil layers. (a1,c1,d1,b1) Comparisons of SOC, ROC, NROC, and C lability between the urban and suburban forests. Error bars represent the standard errors (*n* = 6 for the forest sites, *n* = 3 for the urban and suburban comparison). Different lowercase letters above the bars indicate significant differences at *p* < 0.05.

In the first soil layer (0–10 cm), the SOC content ranged from 19.3 g kg^−1^ to 57.2 g kg^−1^; the significantly highest content was on XT Mountain, the significantly lowest contents were on MF, BY and DH Mountains and the contents on SM and DL Mountains were in between (*p* < 0.05, Fig. 1a). When pooling the six sites as urban and suburban forests, the average SOC content of the urban forests was substantially lower than that of the suburban forests (24.9 ± 2.4 g kg^−1^ vs. 47.1 ± 3.7 g kg^−1^; Fig. 1a1). Similar to the SOC content, the ROC and NROC contents were significantly different among sites in the first soil layer (0–10 cm), with those on XT Mountain being highest and those on MF Mountain being lowest (*p* < 0.05, Fig. 1c,d). As a result, the ROC and NROC contents were substantially lower in the urban forests than in the suburban forests (Fig. 1c1,d1). However, the lability of SOC was not significantly different among sites or between the urban and suburban forests (*p* > 0.05, Fig. 1b,b1). In the second soil layer (10–20 cm), consistent patterns were observed for the SOC, ROC, and NROC contents as well as SOC lability (Fig. 1).

**Figure 2:**
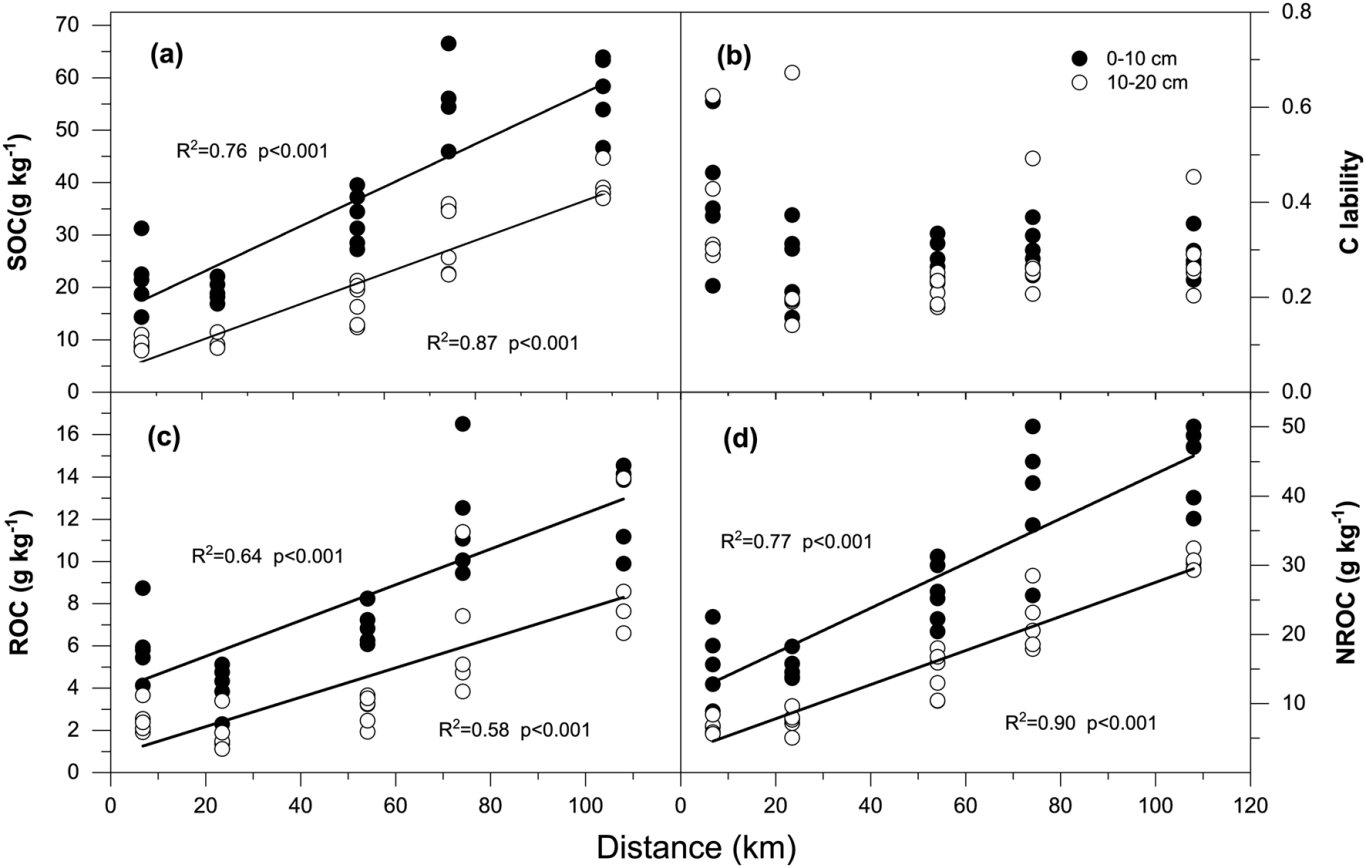
Linear regression relationships between the distance from the urban center and soil C, including the (**a**) total soil organic C (SOC), (**c**) readily oxidizable organic C (ROC) and (**d**) non-readily oxidizable organic C (NROC) concentrations and (**b**) C lability (representing ROC/NROC). The *R*^2^ and *p* values of the linear equations are shown in each panel.

Significant linear relationships were found between the contents of the SOC fractions (including the total SOC, ROC or NROC) and the distance of each forest from the center of the PRD, regardless of the soil layer (*p* < 0.05 for all, Fig. 2a,c,d). The coefficient of determination, *R*^2^, varied from 0.58 to 0.90 in the different fractions, representing high correlations between the contents of the SOC fractions and the distance of forest location from the urban center (Fig. 2a,c,d). The stability of SOC, however, was not significantly related to the distance in either of the two soil layers (*p* > 0.05, Fig. 2b).

### Changes in microbial biomass and microbial community composition

**Figure 3:**
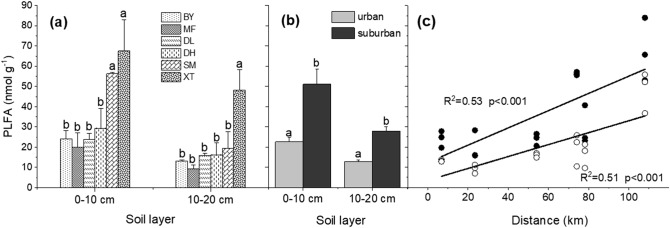
Changes in soil microorganisms in the two soil layers. (**a**) Soil microbial PLFA abundances in every forest site. (**b**) Comparisons of the soil microbial PLFA abundances between the urban and suburban forests. (**c**) Linear regression analysis between the soil microbial PLFA abundances and the distance from the urban center. Error bars represent the standard errors (*n* = 6 for the forest site analysis, *n* = 3 for the urban and suburban comparison). Different lowercase letters above the bars indicate significant differences at *p* < 0.05.

The two-way ANOVA results showed that site and soil layer had significant effects on soil microbial biomass, as indicated by the total PLFA amount in this study (*p* < 0.001), and the interaction between both was also significant (*p* = 0.022, Table 1). Further analysis indicated that the total PLFA amounts ranged from 20.0 ± 7.2 to 67.5 ± 15.5 nmol g^−1^ in the six forests in the 0–10 cm soil layer, with those on MF Mountain being lowest and those on XT Mountain being highest (Fig. 3a). When pooling the urban and suburban forests, the former had significantly lower quantities of total soil PLFAs than the latter (22.6 ± 2.1 nmol g^−1^ in the urban forests vs. 51.1 ± 7.4 nmol g^−1^ in the suburban forests; *p* < 0.05, Fig. 3b). Although the statistical significance among sites differed slightly, the soil microbial biomass pattern in the 10–20 cm soil layer was more or less similar to that in the first soil layer. Furthermore, a positive linear relationship existed between the total soil PLFAs and distance in both soil layers (*R*^2^ = 0.53 and *R*^2^ = 0.51, respectively, *p* < 0.001, Fig. 3c).

**Figure 4:**
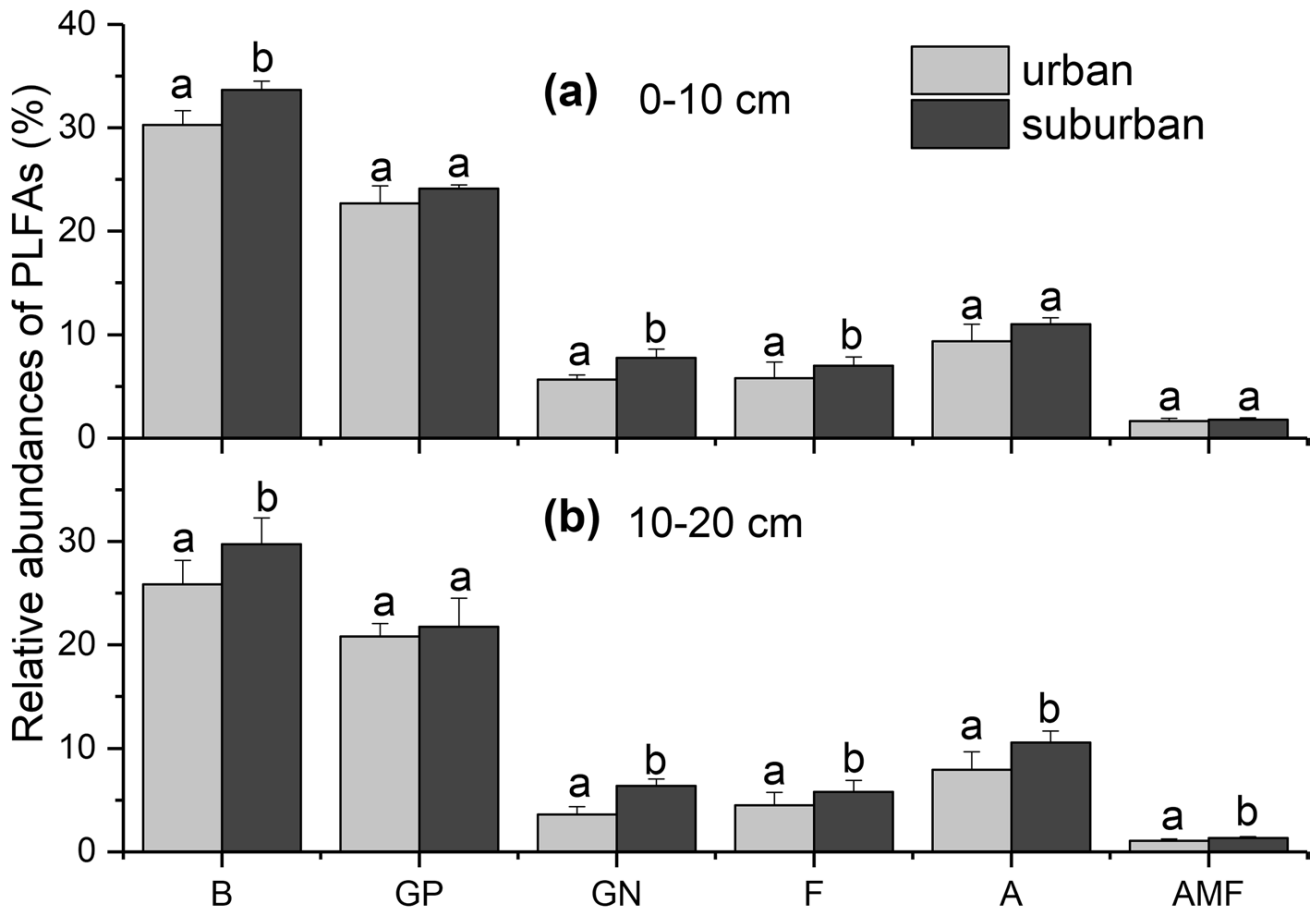
Comparisons of the relative abundances of PLFAs of the different microbial groups between the urban and suburban forests in the two soil layers. B stands for bacteria, GP for gram-positive bacteria, GN for gram-negative bacteria, F for fungi, A for actinomycetes, and AMF for arbuscular mycorrhizal fungi. Error bars represent the standard errors (*n* = 3 for the urban and suburban comparison). Different lowercase letters above the bars indicate significant differences at *p* < 0.05.

The microbial community composition in the urban forests was also significantly different from that in the suburban forests. The relative abundances of several microbial groups were substantially different between the urban and suburban forests (*p* < 0.05, Fig. 4). Briefly, the total and G- bacterial and fungal PLFAs were significantly lower in the urban forests than in the suburban forests in both soil layers (*p* < 0.05). The actinomycetal and arbuscular mycorrhizal fungal PLFAs were significantly lower in the urban forests than in the suburban forests in the second soil layer, but significance was not observed in the first soil layer (*p* < 0.05, Fig. 4). The fungal/bacterial PLFA ratio was not significantly different among sites (*p* > 0.05, Supplementary Fig. S1a), whereas shifts in the bacterial community caused the G+ /G− ratio in the urban forests to be significantly higher than that in the suburban forests (*p* < 0.05, Supplementary Fig. S1b).

### Coupled changes in SOC fractions and microbial community in urban and suburban forests

**Figure 5:**
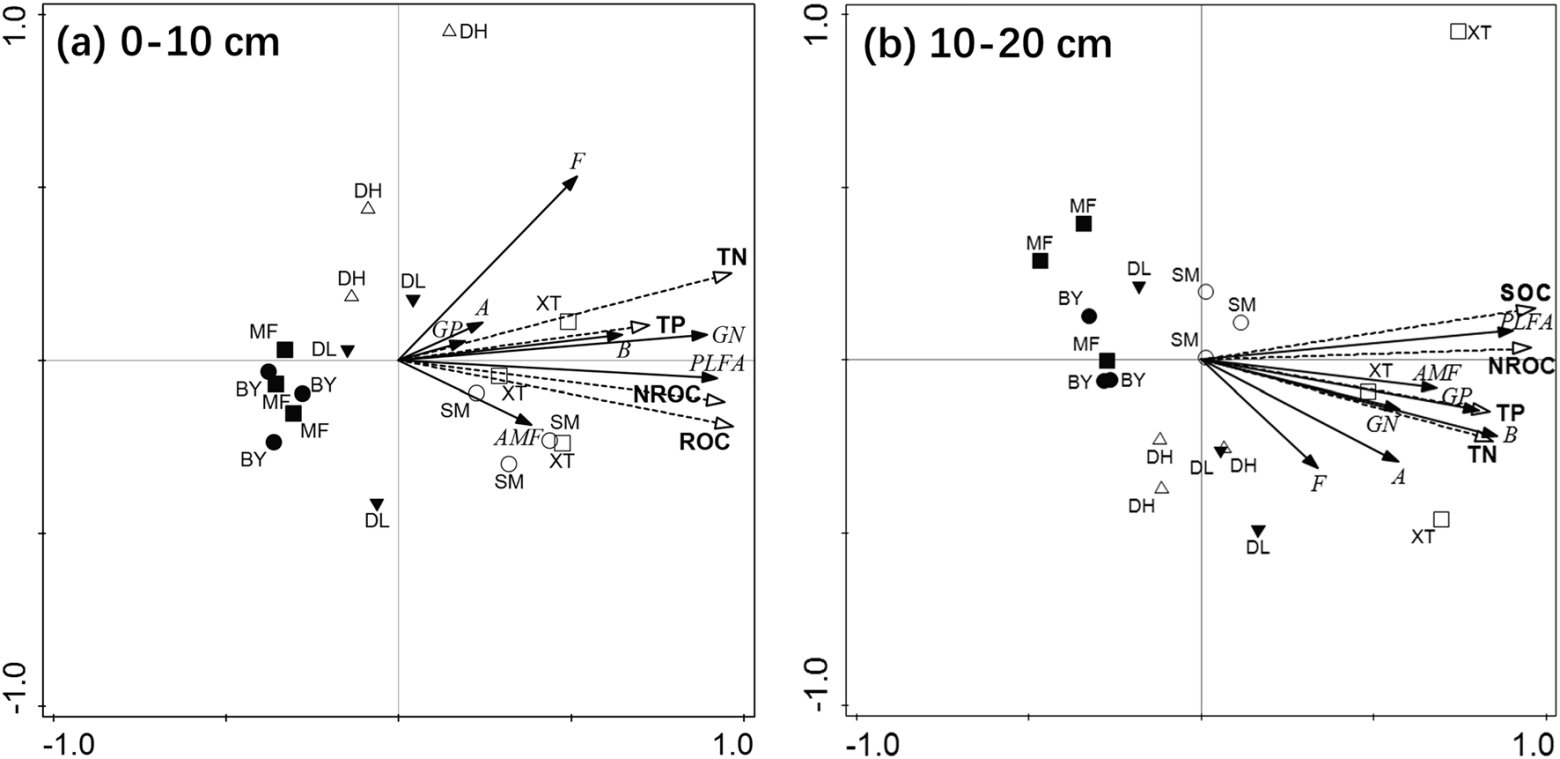
Redundancy analysis between soil C and the soil microorganisms in the 0–10 cm soil layer (**a**) and 10–20 cm soil layer (**b**). The environmental variables involved in the analysis included total soil organic C (SOC), the SOC fractions (ROC represents readily oxidizable organic C, and NROC represents non-readily oxidizable organic C), total nitrogen (TN) and total phosphorus (TP). The species variables included the relative abundances of PLFAs of total bacteria (B), gram-positive bacteria (GP), gram-negative bacteria (GN), fungi (F), actinomycetes (A) and arbuscular mycorrhizal fungi (AMF). The sample number (*n*) was 18 for each of the two soil layers. In both panels, the different symbols represent the different forest sites.

RDA showed that there were significant relationships between the SOC fractions and the abundances of different microbial groups (Fig. 5). The total explanation rates of the soil C fractions to the soil microbial composition were 75.3% and 66.5%, respectively, in the two soil layers, indicating that the variations in the microbial composition were explained by the five soil factors to a great extent (see Supplementary Table S2). The forward selection results showed that the microbial composition was significantly influenced by ROC and TN (*p* < 0.05) in the surface soil layer, while total phosphorus (TP) and NROC had negligible conditional effects on the soil microbial composition in comparison. In the subsurface soil layer, SOC was the primary influencing factor, having both marginal effects and conditional term effects (*p* < 0.05) (see Supplementary Table S2).

In detail, SOC, ROC, and NROC were positively related to the total PLFAs in both soil layers. Likewise, the relative abundances of bacterial PLFAs, including both G+ and G− bacteria, were positively related to TN and TP. Compared with SOC and its fractions, TN was more closely correlated with the fungal and actinomycete PLFAs. The sample distribution, in which the urban forest sites were clearly isolated from the suburban forest sites along the first axis, also indicated the differences in the soil C contents and soil microbial PLFA abundances between the urban and suburban forests. Furthermore, the different forest sites were clearly separated by their PLFA-derived soil microbial community compositions, and the results were consistent in the two soil layers. Interestingly, the suburban forest sites were scattered more than the other three sites that represented urban forests, which could be inferred that the impact induced by environmental change on the forest soil microbial composition in the urban area was potentially more significant than that in the suburbs (Fig. 5).

## Discussion

A significantly lower SOC content in the urban forests than in the suburban forests was observed in this study. This pattern was consistent with the research in subtropical forests reported by Chen et al.^30^ but differed from studies along a New York City urban–rural gradient^36,37,40^. According to field investigations (see Supplementary Table S1) and previous studies^30^ in subtropical forests, we inferred that a greater C pool in the suburban forests was probably linked to higher vegetation productivity, as well as more fine root biomass^30^, which were considered to be significantly and linearly related to vegetation productivity^41^. The accumulation of aboveground litter on the soil surface or through root litter and exudates from the plant is highly important to the soil C inputs^42,43^. Meanwhile^30^, litterfall P content was found to be significantly lower in urban forests than in suburban forests (see Supplementary Table S1), which may affect litter decomposition and then the C inputs, by strengthening the nutrition restriction on microbial activities^44–46^. Notably, stand age and forest coverage should not have resulted in differences in SOC content. Despite being the same in stand age and forest coverage between some of the investigated urban and suburban forests, the SOC contents in the surface soil of the BY and SM forests differed significantly (21.67 g kg^−1^ vs. 51.62 g kg^−1^, respectively; Fig. 1a).

In further analysis, the lower content of SOC in the urban forests was derived from the reduction in both the labile and nonlabile SOC fractions (Fig. 1c,d), which was confirmed by the significant relationship between the SOC content and ROC and NROC contents (Fig. 5). The labile SOC fraction, with a small size and rapid turnover, is a direct reservoir of readily available nutrients^47^. Soil microorganisms tend to utilize the substrate of this fraction^48^, i.e., ROC, representing the chemically labile SOC fraction^49^. Relative to urban forests, higher ROC content in suburban forests is closely related to changes in microbial biomass and plant-derived C inputs^50,51^ (Fig. 5). Conversely, the nonlabile fraction turns over slowly and is more resistant to microbial decomposition. Nonlabile SOC fraction accumulation has a great impact on soil C sequestration and the stability of the C pool^15^. It was confirmed that higher temperatures in urban areas led to the consumption of large amounts of nonlabile SOC fractions^30,38^. NROC represents the chemically nonlabile SOC fraction and an important component of the SOC content^49^. Although the C lability and C/N ratio were not significantly changed by urbanization, the reduction in both ROC and NROC contents suggests that urbanization could not only affect the soil C pool but also the SOC composition, and this finding may impair soil C accumulation (Fig. 1, Supplementary Table S1).

**Figure 6:**
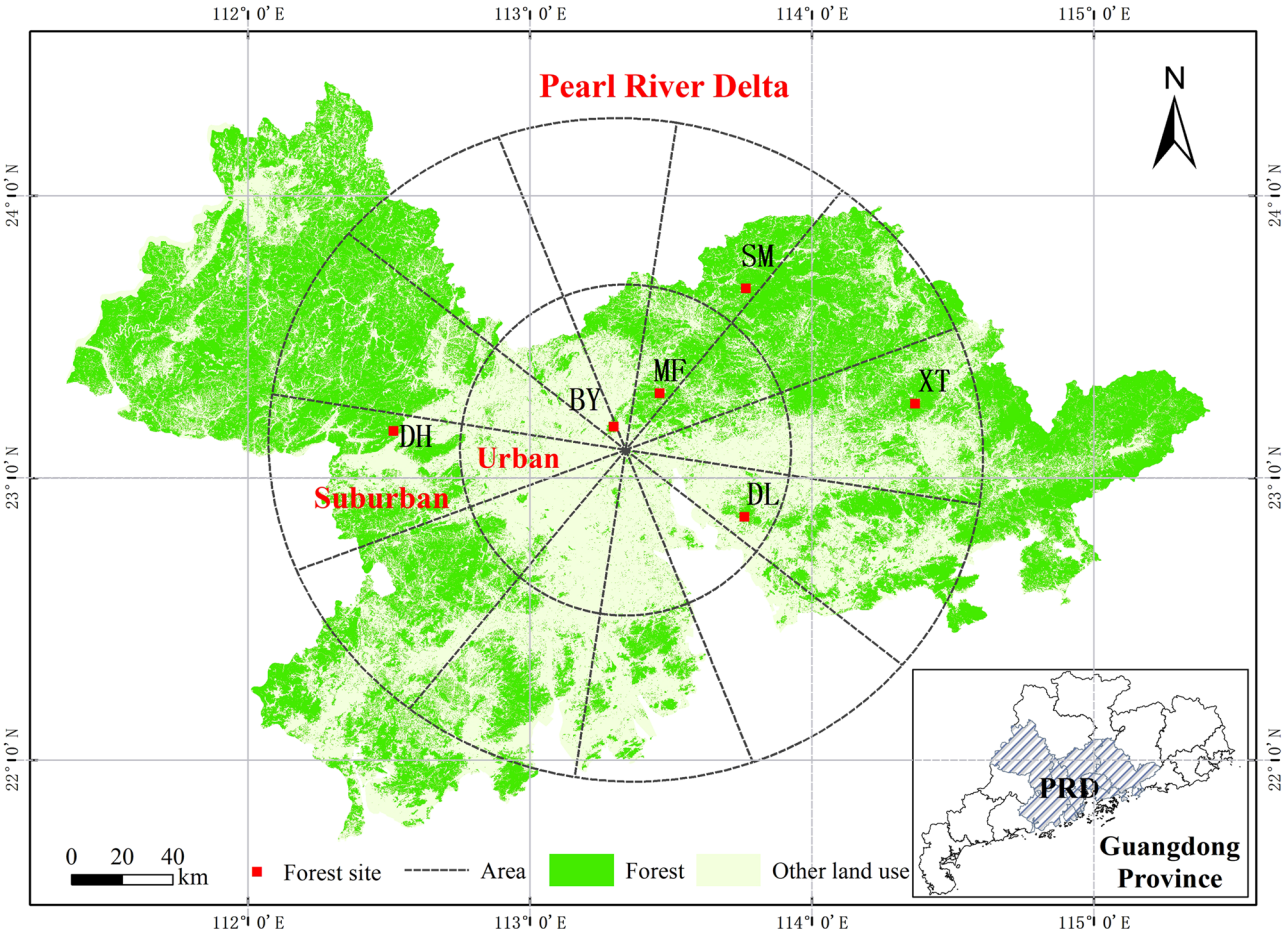
The geographical location of the study sites in the Pearl River Delta (PRD), in Guangdong Province of southern China. A total of six forest sites were selected; BY, MF, and DL were the urban forests (0–65 km), and DH, SM, and XT were the suburban forests (65–130 km). The map was created by ArcMap 10.2 software (Environmental Systems Research Institute, Inc. (ESRI), US).

Furthermore, the direction and magnitude of how urbanization affects soil microbial biomass are inconclusive in subtropical forests^30,52,53^. In this study, we observed that the total amount of PLFAs in the suburban sites was twice more than that in urban sites (Fig. 3). Total PLFAs are frequently used to indicate soil microbial biomass because they are often well correlated with soil microbial biomass C (MBC, as determined by the fumigation-extraction method)^54^, which is regarded as the chief component of the labile C pool^17^. In consideration of the high correlation between soil total PLFAs and soil nutrient factors in both soil layers, we inferred that nutrition restriction is one of the main reasons for the different responses of soil microorganisms to urbanization between urban sites and suburbs, especially in the surface soil, the layer with abundant litter and roots and large microbe aggregations (Figs. 3, 6). Severer C limitation in urban forests than suburban forests may cause the reduction of soil microbial biomass^34,55^. A large proportion of plant material is transformed from soil microorganisms into soil C on the one hand and^56^, on the other hand, microbial products contribute greatly to stable C^25,57,58^. Therefore, the SOC content exhibited a decrease in concentration in the urban forests, which was likely derived from lower microbial biomass and thus affected both the labile C fraction and nonlabile C fraction.

The microbial contribution to soil C depends on both microbial activity and community structure^1,21^. Changes in microbial community structure probably altered C sequestration because different microbial categories, such as fungi and bacteria, generally utilized different C sources^24,59^, which was reflected specifically in our results that various microbial groups showed different sensitivities to urbanization-induced environmental change (Figs. 4, 6). In detail, urbanization significantly decreased the abundance of fungi and bacteria, especially G-negative bacteria, in both soil layers (Fig. 4). Fungi can decompose a wide variety of complex and recalcitrant plant-derived materials to produce organic C; similarly, G− bacteria prefer to utilize the C of fresh plant materials (such as plant debris and root exudates) as C sources, while there was more decomposition of soil C by G+ bacteria because of high soil C assimilation^60,61^. These results may be an explanation of lower C inputs and thus a decrease in the labile C fraction in the investigated urban forests. Although there is a small proportion of fungal PLFAs in the total PLFAs than bacteria, dead fungal biomass is particularly important as a transient N pool because it represents a unique substrate that is typically N-rich^62^. The fungi and mycorrhizae play a mediating role in the C flow from primary producers into the soil, and the significant correlations between TN and fungal PLFAs (Fig. 5) demonstrate that a reduction in the fungal population may affect the soil N pool in the urban forests (see Supplementary Table S1). It is noteworthy that fungi, especially mycorrhizal fungi, are sensitive to disturbance (such as changes in soil physical and chemical properties) in the remnant vegetation in urban areas^26^.

Our observation that the G+ /G− bacterial PLFA ratio increased significantly in the urban forest in both soil layers (see Supplementary Fig. S1b) further confirmed the impact of urbanization on the bacterial composition. It has been widely proven that fungal products are more chemically resistant to decay than bacterial products, and fungal products contribute significantly to soil C storage for their involvement in promoting soil aggregation^57,63,64^. However, it was also reported that bacteria contained more alkyl C but less O-alkyl C than fungi, and thus, a high abundance of soil G+ bacteria was linked to the high stable soil C content because of the teichoic acids in the G+ bacterial cell wall^56^. Although there was a significant decrease only in fungal PLFAs and G− bacterial PLFAs rather than G+ bacterial PLFAs, we noticed a decreasing trend of G+ bacteria in the urban forests. In consideration of the absolute proportion of G+ bacteria, it was necessary to further confirm its response to urbanization for a longer time in this bacterial-dominated microbial community in subtropical forests. Actinomycetes can also form humus-like polymers that are favorable to soil C sequestration^65^. In the urban forests, significant declines in arbuscular mycorrhizal fungal and actinomycete PLFAs in the subsurface soil layer, where nutrient limitation is more serious, also require more attention (Fig. 4). In general, the above changes in microbial community composition are important causes influencing nonlabile SOC fraction in urban sites.

The changes in the SOC fractions and soil microbial community composition were observed to be significantly related to the distance of the study sites from the urban center (Figs. 2, 3). The translocation from urban and suburban regions represents a change in a combination of several ecological factors and the soil microenvironment^37^. Lower soil pH, increased atmospheric N deposition, and CO_2_ enrichment have been reported to be more serious in urban forests than in suburban forests^4,66–68^, and the urban heat island effect and atmospheric acid deposition are also not allowed to ignore^9,69^. N deposition, CO_2_ enrichment, and warming can significantly increase labile SOC and promote soil respiration and its sensitivity to warming by stimulating plant C inputs^13,70,71^; however, they cause more soil C loss, which is likely due to the “priming effect”, i.e., the short-term changes in the turnover rate of SOM induced by addition to the fresh organic matter in the soil^72,73^. Furthermore, the interactive effect between the elevated atmospheric CO_2_ and low water availability has been proven to be essential in shaping the soil microbial community composition^74^. Likewise, warming can also change the microbial community structure and then affect the decomposition of labile and nonlabile SOC fractions^58^. Soil mineralization promoted by “priming effects” may be related to the increases of labile SOC content and specialized microorganisms in decomposing fresh organic matter^73,75,76^. Although the responses of SOC and its fractions to urbanization had been studied in this paper, further studies are needed to combine these results with SOC mineralization experiments to accurately predict how soil C sequestration responds to urbanization.

## Conclusions

In the southern subtropical evergreen broadleaved forest, the significant reduction of SOC content and soil microbial biomass was probably attributed to soil nutrient restriction and potential differences in C inputs in the urban remnant forests. SOC and soil microbes interact and have feedback with each other. On the one hand, C limitation in urban forests, especially in the subsurface soil layer, is therefore a plausible reason for explaining the differences in the soil microbial biomass between urban forests and suburban forests. The reductions in both the labile and nonlabile SOC fractions suggested that urbanization could affect not only soil C pool but also the SOC composition, which indicated the low quantity and quality of SOC in the urban forests, and consequently impacted microbial community abundance and structure. On the other hand, we further found shifts in the microbial community structure in both soil layers, which were mainly represented by significant decreases in the relative abundances of fungi and bacteria, especially G− bacteria. This observation was found to cause the loss of soil C pools by affecting C inputs and promoting the decomposition of SOC; therefore, the coupled changes in soil SOC and soil microorganisms induced by urbanization would potentially have adverse effects on soil C sequestration in subtropical forests. The findings could help predict the possible future impacts of persistent climate change on forest ecosystems, especially the effect on soil C sequestration, and the accompanying change in soil microorganisms. Further study should focus on the mechanism of how changes in the ecological factors caused by urbanization reduce the SOC content.

## Material and methods

### Study region and site description

The study was performed in the PRD, South China (21° 17.6′–23° 55.9′ N, 111° 59.7′–115° 25.3′ E). This region has a typical subtropical monsoon climate; the highest rainfall and temperatures occur in July, with annual precipitation and air temperatures of approximately 1800 mm and 22 °C, respectively. The soil in all the selected forest sites was shallow ultisol overlying sandstone and shale bedrock. The soil pH was 3.9 and 4.0 in the topsoil and subsurface soil (0–10 cm and 10–20 cm soil layers, respectively). Subtropical monsoon broadleaved forests are widely distributed in the region as zonal vegetation.

The PRD economic region includes nine cities at the prefecture-level in which Guangzhou (the capital of Guangdong Province) is the dominant city in the PRD and is considered to be a provincial political, economic, and cultural center. Considering the situation of construction activities, land use, population, and energy consumption (Guangdong Statistical Yearbook 2019), there is an urban development intensity gradient in the PRD, and apparently, Guangzhou can be defined as the core of the PRD based on the intensity of socioeconomic activities, i.e., the urban center in the urban–rural gradient in this study^30,77^. Based on the distance from the urban center, we first divided the study region into urban areas and suburban areas. As a result, two concentric circles were extended outward from the core of the PRD: 0–65 km was considered the urban area, and 65–130 km was considered the suburban area^30,66,67^. We further divided each class into 12 subzones with equal areas, and then three subzones were chosen randomly. Considering that the urbanization pattern in this region was not always linear^30^, we combined the changes in land use for forestry and construction uses by analyzing the Landsat 5 TM satellite images and the Landsat 8 OLI satellite images to confirm the sample location. To ensure the limited effect of human activities in the preselected area near the city, the populations of prefecture-level cities in the PRD were also considered in the division. After that, according to a field investigation, six evergreen broadleaved forests were selected that remained unmanaged and had no disturbance after planting. There were three forests in the urban area (Baiyunshan, Maofengshan, and Dalingshan, abbreviated BY, MF, and DL, respectively) and three forests in the suburban area (Dinghushan, Shimen National Forest Park, and Xiangtoushan, abbreviated DH, SM, and XT, respectively) (Fig. 6). The vegetation and soil properties of the studied forests are listed in Supplementary Table S1.

### Soil sample collection and analyses

In July 2015, five plots (5 m × 5 m square) were selected in each of the six sites, and the minimum distance between any two plots was 10 m. To ensure comparability among the forests, several criteria were used to select the plots: the elevations of all selected plots were between 200 and 400 m, the slope was 20°–30°, and the slope aspect was southeast. The following considerations should also be given to the selection: the forest community was a typical evergreen broadleaved forest; each plot was located 1 km from the main road, and the area was not less than 1 ha; and there was no direct or serious human disturbance after planning, including fire, logging, and fertilization. In total, 30 plots were chosen in all six forest sites. We collected litter and soil samples in every subplot (60 cm × 60 cm square) from the center of the selected plot (5 m × 5 m square) to avoid edge effects. The litter samples were sorted to remove fresh leaves and semidecomposed leaves and then dried at 40 °C for a week to a constant weight to determine the litterfall biomass. Soil samples were collected at depths of 0–10 cm and 10–20 cm using a soil corer (3 cm inner diameter). The samples were mixed to obtain a composite sample for each of the three soil layers in every plot (5 m × 5 m square).

All composite soil samples were sieved to pass through a 2 mm mesh after visible stones, roots, and plant residues were removed. Each soil sample was then divided into two portions. One portion was stored in a refrigerator at − 4 °C for the PLFA analysis, and the other portion was air-dried for the physicochemical measurements. The total SOC and TN were determined with a Vario EL elemental analyzer (Elementar, Hanau, Germany). TP was determined using the colorimetric method^78^. The SOC, TN, and TP contents in the organic layers and mineral layers, as well as their stoichiometric ratios, are shown in Table 1.

### SOC fractions

In this study, SOC was separated into two fractions, i.e., the labile and nonlabile SOC fractions. To determine the SOC fractions, physical, chemical, and biological methods and ^13^C nuclear magnetic resonance (NMR) techniques have been widely used^17,49,50^. We used the chemical oxidation method because ROC is more sensitive than particulate organic carbon (POC) and MBC when assessing management practices or environmental change^50,51^. KMnO_4_ oxidation was used to separate these two SOC fractions, in which the labile SOC fraction was indicated by ROC, and the nonlabile SOC fraction was indicated by NROC^49^. In detail, the air-dried soil samples were further ground to pass through a 149 μm mesh before the analysis. Soil containing 15–30 mg of C was weighed into 100 mL plastic centrifuge tubes, and 25 mL of a 333 mmol L^−1^ KMnO_4_ solution was added^49^. The tubes were shaken for 1 h at 250 r min^−1^ and centrifuged for 5 min at a speed of 2000 r min^−1^. One milliliter of the supernatant was then diluted 250 times with deionized water, and the color development was read at a wavelength of 565 nm on a UV spectrophotometer (UV-1750, Shimadzu International Trading (Shanghai) Co., Ltd., China). Four controls without soil samples were treated in the same manner. The soil ROC concentration was calculated based on differences in consumption of the KMnO_4_ solution between the samples and controls and assuming that 1 mmol of KMnO_4_ was consumed in the oxidation of 9 mg C. The difference between the SOC and ROC concentrations was calculated as the soil NROC concentration, and the ratio of ROC to NROC indicated the C lability^15,49^.

### PLFA analysis

We examined the soil microbial community with phospholipid fatty acid (PLFA) analysis; this method has been widely used for measuring soil microbial amounts and categories because of its low cost and sensitivity at the community level^79^. The experimental operation referred to the method proposed by Bossio et al.^80^, with minor modifications. Briefly, for each sample, 8 g of freeze-dried soil was extracted in chloroform–methanol-citrate buffer (1:2:0.8 v/v/v), and the phospholipids in the organic phase were then separated and collected using a silica column (500 mg, ANPEL Laboratory Technologies Inc., Shanghai, China). Neutral lipids, glycol lipids, and polar lipids were eluted from the column with chloroform, acetone, and methanol; then, the polar lipid fraction was transmethylated into fatty acid methyl esters using 1 mL of 0.2 mol methanolic KOH. The fatty acids were analyzed with an Agilent 7890 gas chromatograph (GC) equipped with a Sherlock microbial identification system (version 6.2, MIDI Inc., Newark, DE, US). Methyl nonadecanoate fatty acid (19: 0) was used as an internal standard to confirm the individual PLFA contents (nmol g^−1^ soil) and relative abundances of the microbial groups (mol%).

In the analysis, 15:0, 17:0, i14:0, i15:0, a15:0, i16:0, i17:0, a17:0, 16:1ω7c, cy17:0 and 18:1w7c were considered to be the bacterial PLFAs, while 18:1w9c and 18:2w6c were indicative of the fungal PLFAs. The gram-positive (GP) bacteria were the sum of the iso- and anteiso-fatty acids, while the gram-negative (GN) bacteria were the sum of the cyclopropane fatty acids, monounsaturated fatty acids and hydroxyl fatty acids. The PLFAs 10Me16:0, 10Me17:0, and 10Me18:0 represented actinomycetes, and 16:1 w 5c was regarded as an indicator of arbuscular mycorrhizal fungi. The ratio of fungal to bacterial PLFAs (F/B) and the ratio of GP to GN bacteria (G+/G−) were calculated to indicate the soil microbial community composition^81^.

### Statistics

The normality of the data was tested using the Shapiro–Wilk method. Homogeneity of variances among groups was tested with Levene’s test to ensure the equality of error variances. One-way ANOVA with Scheffe post hoc multiple comparisons was conducted to compare the means of the SOC fractions and C lability among the sites when the equal variances assumption was met; otherwise, the Games–Howell method was used. Multiple comparisons with the Tukey method were performed to assess the significance of the sites regarding the total PLFA amount. Two-way ANOVA was used to determine the effects of site and soil layer and their interaction on the SOC fractions and total PLFA amount. An independent-sample *t*-test was used to detect significant differences in litterfall production, soil nutrients and ratios, SOC fractions, total PLFA amount, and soil microbial community composition between the urban and suburban forests. To confirm whether distance caused the differences, linear regression analysis was used. Redundancy analysis (RDA) was used to determine coupled changes in the SOC fractions and microbial community. Differences were considered significant at *p* < 0.05. All analyses were conducted with IBM SPSS statistics 20 software (IBM Corp., New York, US).

## Supplementary information

Supplementary Information.

## Data Availability

The Landsat 5 TM image and Landsat 8 OLI satellite remote sensing image data are available for download at the Geospatial Data Cloud (https://www.gscloud.cn); other research data supporting this article are available to anyone for research by request to Xiaomei Chen.
