# Supplementary material for: Coupled changes in soil organic carbon fractions and microbial community composition in urban and suburban forests
Source: Sci Rep. 2020 Sep 28;10:15933. doi: 10.1038/s41598-020-73119-8 (PMC7522236; doi:10.1038/s41598-020-73119-8)

Supplementary information

Coupled changes in soil organic carbon fractions and microbial community composition in urban and suburban forests

Xueying Zhang[1], Xiaomei Chen[1]*, Muying Liu[1], Zhanying Xu[1] and Hui Wei[2]

[a] School of Geographical Sciences, Guangzhou University, Guangzhou 510006, China.

[b] College of Natural Resources and Environment, South China Agricultural University, Guangzhou 510483, China.

Corresponding author: Xiaomei Chen

E-mail address: chxm-009@163.com

Postal address: School of Geographical Sciences, Guangzhou University, 230 Wai Huan Xi Road, Guangzhou Higher Education Mega Center, Guangzhou 510006, P. R. China

Supplementary Table 1

Initial vegetation and soil properties in the studied forests.

<table>
<tr><th colspan="2" rowspan="2">Site</th><th colspan="3">Urban</th><th colspan="3">Suburban</th></tr>
<tr><th>BY</th><th>MF</th><th>DL</th><th>DH</th><th>SM</th><th>XT</th></tr>
<tr><td colspan="2">Location</td><td>113.30°E, 23.19°N</td><td>113.47°E, 23.30°N</td><td>113.81°E, 22.87°N</td><td>112.56°E, 23.17°N</td><td>113.78°E, 23.65°N</td><td>113.78°E, 23.31°N</td></tr>
<tr><td colspan="2">Stand age /y</td><td>>60</td><td>>50</td><td><40</td><td>approximately 400</td><td>>70</td><td><60</td></tr>
<tr><td colspan="2">Forest coverage</td><td>90%</td><td>80% to 85%</td><td>75%</td><td>> 90%</td><td>> 90%</td><td>80%</td></tr>
<tr><td colspan="2">Dominant species (Arbor)</td><td>Schima superba</td><td>Schima superba</td><td>Schima superba</td><td>Schima superba; Castanopsis chinensis; Cryptocarya chinensis et al.</td><td>Schima superba; Castanopsis chinensis; Machilus chinensis et al.</td><td>Rhodoleiac hampionii Hook.f.</td></tr>
<tr><td rowspan="7">Organic layer</td><td>Litterfall biomass /g·m^{-2}</td><td colspan="3">1022.336 ± 179.347 a</td><td colspan="3">941.905 ± 304.383 a</td></tr>
<tr><td>C /g·kg^{-1}</td><td colspan="3">45.285 ± 3.992 a</td><td colspan="3">46.380 ± 3.265 a</td></tr>
<tr><td>N /g·kg^{-1}</td><td colspan="3">4.538 ± 0.317 a</td><td colspan="3">5.593 ± 1.316 a</td></tr>
<tr><td>P /g·kg^{-1}</td><td colspan="3">0.164 ± 0.021 a</td><td colspan="3">0.235 ± 0.052 b</td></tr>
<tr><td>C/N</td><td colspan="3">11.794 ± 2.987 a</td><td colspan="3">8.277 ± 2.373 a</td></tr>
<tr><td>N/P</td><td colspan="3">25.138 ± 4.541a</td><td colspan="3">23.942 ± 6.396 a</td></tr>
<tr><td>C/P</td><td colspan="3">299.524 ± 106.423 a</td><td colspan="3">196.571 ± 84.387 b</td></tr>
<tr><td rowspan="4">Mineral layer</td><td>Soil layer</td><td>0-10 cm</td><td colspan="2">10-20 cm</td><td>0-10 cm</td><td colspan="2">10-20 cm</td></tr>
<tr><td>C /g·kg^{-1}</td><td>24.910 ± 2.390 a</td><td colspan="2">12.514 ± 0.763 a</td><td>47.067 ± 3.700 b</td><td colspan="2">27.596 ± 1.741 b</td></tr>
<tr><td>N /g·kg^{-1}</td><td>1.5075 ± 0.176 a</td><td colspan="2">1.128 ± 0.319 a</td><td>3.016 ± 0.209 b</td><td colspan="2">2.103 ± 0.31 b</td></tr>
<tr><td>P /g·kg^{-1}</td><td>0.129 ± 0.002 a</td><td colspan="2">0.115 ± 0.004 a</td><td>0.195 ± 0.005 b</td><td colspan="2">0.16 ± 0.01 a</td></tr>
</table>

C/N	16.862 ± 1.244 a	12.565 ± 1.719 a	15.549 ± 0.381 a	13.121 ± 1.625 a
N/P	12.132 ± 0.606 a	9.317 ± 1.684 a	16.601 ± 1.076 b	15.547 ± 2.821 b
C/P	199.311 ± 6.757 a	104.998 ± 16.992 a	254.991 ± 17.923 a	192.05 ± 16.787 b

The values of the soil properties are the mean ± the standard deviation (n=3). Significant differences (at the p<0.05 level) between the urban and suburban forests are indicated by different lowercase letters. C represents total soil organic C, N represents total nitrogen, and P represents total phosphorus. C/N, N/P, and C/P represents the C, N and P stoichiometry between two elements.

Supplementary Table 2

Marginal and conditional effects of the soil C fractions on the soil microbial community composition obtained from the forward selection summary in the redundancy analysis (RDA).

0-10 cm	Marginal effects /%	Conditional term effects /%	*F*	*P*	10-20 cm	Marginal effects /%	Conditional term effects/%	*F*	*P*
ROC	66.9	88.8	32.3	0.002	SOC	58.7	88.2	22.7	0.002
TN	6.5	8.6	3.7	0.022	TP	3.9	5.8	1.5	0.194
TP	0.9	1.2	0.5	0.67	TN	2.5	3.8	1	0.37
NROC	1	1.3	0.5	0.632	NROC	1.5	2.3	0.6	0.63
Total explanation rate of the environmental variables to the response variables：75.3%					Total explanation rate of the environmental variables to the response variables：66.5%				

Supplementary Figure 1 Comparisons of the fungal PLFA content to the bacterial PLFA content ratio (F/B) and gram-positive bacterial PLFA content to the gram-negative bacterial PLFA content ratio (G+/G-) between the urban and suburban forests in the two soil layers. Error bars represent the standard errors (n=3 for the urban and suburban comparison). Different lowercase letters above the bars indicate significant differences at p<0.05.

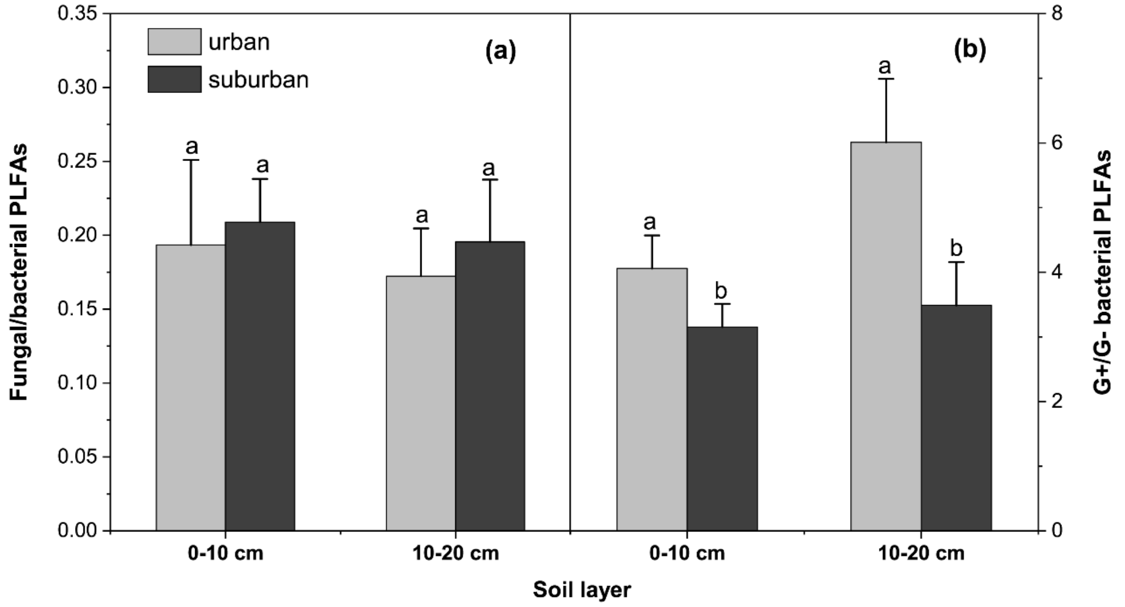

Supplement: Supplementary file 1 — Supplementary Information. [file 41598_2020_73119_MOESM1_ESM.pdf]
